# Sustainable development goals and ending ECC as a public health crisis

**DOI:** 10.3389/fpubh.2022.931243

**Published:** 2022-10-18

**Authors:** Ankita Saikia, Jagadeesan Aarthi, MS Muthu, Sneha S. Patil, Robert Prashanth Anthonappa, Tarun Walia, Moayad Shahwan, Peter Mossey, Monica Dominguez

**Affiliations:** ^1^Department of Pediatric Dentistry, Centre for Early Childhood Caries Research (CECCRe), Sri Ramachandra Institute of Higher Education and Research, Chennai, Tamil Nadu, India; ^2^Department of Pediatric and Preventive Dentistry, Madha Dental College and Hospital, Chennai, India; ^3^Dental School, Oral Developmental and Behavioural Sciences, University of Western Australia, Perth, WA, Australia; ^4^Centre of Medical and Bio allied Health Sciences Research, Ajman University, Ajman, United Arab Emirates; ^5^Dundee Dental School, University of Dundee, Dundee, United Kingdom; ^6^Global Oral Health Programs, Smile Train Head Office, New York, NY, United States

**Keywords:** SDG, early childhood caries, oral health, sustained anticipatory guidance, child health, healthcare workers, infant oral care, first dental visit

## Abstract

Early Childhood Caries (ECC) remains a global issue despite numerous advancements in research and interventional approaches. Nearly, 530 million children suffer from untreated dental caries of primary teeth. The consequences of such untreated dental caries not only limit the child's chewing and eating abilities but also, significantly impact the child's overall growth. Research has demonstrated that ECC is associated with nearly 123 risk factors. ECC has also been associated with local pain, infections, abscesses, and sleep pattern. Furthermore, it can affect the child's emotional status and decrease their ability to learn or perform their usual activities. In high-income countries, dental care continues to endorse a “current treatment-based approach” that involves high-technology, interventionist, and specialized approaches. While such approaches provide immediate benefit at an individual level, it fails to intercept the underlying causes of the disease at large. In low-income and middle-income countries (LMICs), the “current treatment approach” often remains limited, unaffordable, and unsuitable for the majority of the population. Rather, dentistry needs to focus on “sustainable goals” and integrate dental care with the mainstream healthcare system and primary care services. Dental care systems should promote “early first dental visits,” when the child is 1 year of age or when the first tooth arrives. The serious shortages of appropriately trained oral healthcare personnel in certain regions of the world, lack of appropriate technologies and isolation of oral health services from the health system, and limited adoption of prevention and oral health promotion can pose as critical barriers. The oral health care systems must focus on three major keystones to combat the burden of ECC−1. Essential oral health services are integrated into healthcare in every country ensuring the availability of appropriate healthcare accessible and available globally, 2. Integrating oral and general healthcare to effectively prevent and manage oral disease and improve oral health, 3. Collaborating with a wide range of health workers to deliver sustainable oral health care tailored to cater to the oral health care needs of local communities.

## Introduction

The Sustainable Development Goals (SDGs) framework, first launched in 2015 during the UN General Assembly, includes 17 universal goals and 169 targets, while the sustainable development agendas are envisioned to be accomplished by 2030 (1). Of these SDGs, the health goal (SDG3) is to focus on good health and well-being (1, 2). World Health Organization (WHO) defines health as “a state of complete physical, mental and social well-being and not merely the absence of disease or infirmity.” Good oral health is complementary to good general health. Approximately 3.5 billion people worldwide are affected by oral disease, of whom 530 million are children (3). However, despite its high prevalence, oral health remains primarily neglected and is neither included under Universal Health Coverage (UHC) of global health (4), nor mentioned in SDG3. Similarly, chronic diseases are the leading cause of hospitalization, accounting for nearly 46% of the global disease burden (5, 6). Moreover, the evolving landscape of chronic diseases and their co-existing risk factors levitate each other, thus sparking global concern (6–8). It is to be noted that dental caries remains the most common chronic disease of humans (9).

Early Childhood Caries (ECC) is a common chronic disease of childhood (10), and its prevalence is five times higher than asthma and seven times more than hay fever (11, 12). ECC disproportionally affects the economically deprived families and vulnerable population groups (13). Limited infrastructure, inadequate workforce, and significant barriers to dental care are social determinants related to poor oral health (14, 15), resulting in millions of children with untreated caries (16). Subsequently, the negative consequences, namely, pain, infections, abscesses, disturbed sleep and eating patterns, loss of school days, reduced activity, visits to the emergency departments, and hospitalisations, significantly impair the child's and their family's overall health-related quality of life (9, 17). Moreover, poor dental health in childhood frequently results in poor dental health in adulthood (10, 17, 18).

Despite ECC being largely preventable, its increasing prevalence continues to raise concern. The coinciding inequalities in oral health, both within and between countries, high cost of dental treatment and competing health priorities with available resources to address are policy makers' concerns (19). Moreover, in many world regions, the uneven distribution of oral healthcare personnel and a lack of coherent stakeholders and policymakers are barriers to implementing preventive approaches (10, 11, 15). Therefore, population-wide strategies with sustainable maneuvers are critical to evaluating practical, sustainable approaches to reduce the ECC burden globally. Nevertheless, no policy/recommendations emphasize on SDGs of ECC and their targets. Hence, this paper highlights three significant strategies to combat the burden of ECC, namely. (i) Integration of oral health services into primary health care centers (ii) amalgamating oral and general healthcare for better patient care and (iii) Multidisciplinary approach collaborating with wide range of health care workers.

## Methods

A PubMed and google scholar search was conducted using the following keywords: ECC, Sustainable Developmental Goals, SDG, Oral Health and Policy, fields: all; without limits on the year of publication. Additionally, websites of the World Health Organization (WHO), FDI and National Immunization Schedules (NIS) were reviewed.

### Integration of oral health services into primary health care centers

Primary Health Care (PHC) is an all-inclusive society approach toward health and well-being. It focuses on people's needs along with continued health promotion, disease prevention, treatment, rehabilitation and palliative care in concordance with people's day-to-day environment (20, 21). The vision of PHC is to achieve Universal Health Coverage (UHC) and SDGs. The various determinants of health and interlinked aspects of physical, mental and social health, well-being are addressed by PHC. This indicates the necessity of a comprehensive PHC workforce which works in multitudinal directions (22) including oral health.

#### Integrating immunization schedule with early dental visits

In order to achieve the SDG target of early diagnosis, it is imperative to work in concordance with medical personnel and integrate oral health monitoring at each stage of primary tooth eruption with immunization schedule. Most governing bodies of children such as AAPD, the American Academy of Pediatrics (AAP), American Dental Association (ADA) recommend the children to have their first dental visit within 1 year of their life (23). Unfortunately, oral health is not part of pediatric primary healthcare (22, 23), and efforts for unification are often a low priority (24, 25), causing oral health disparities in children. Therefore, inter professional collaborative efforts among health professionals are critical for ECC prevention. Furthermore, such initiatives will address this public health crisis by ensuring all infants and toddlers to have access to dental screenings that are synchronized with vaccination schedules, and allow age appropriate counseling, and preventive procedures (24). Establishing an integrated periodic dental screening in line with immunization schedule could be instrumental in implementing the first dental visit by year one or as soon as the first tooth erupts into the oral cavity (Table 1).

**Table 1:** Integrating vaccination schedule with eruption of various primary teeth.

**WHO immunization schedule**	**Vaccination schedule (NIS, India)**	**Teeth to be screened**	**Implementation of SDG Target**
9 or 12 months Measles, rubeola/ 9–18 months Td/DT containing vaccine Pneumococcal conjugate booster	9–12 Months Measles, rubeola, PCV Booster	Lower central incisor Upper central and lateral incisor	First dental visit SAG Oral health education to parents Early diagnosis of enamel defects for White Spot Lesions (WSL).
12–23 months DTP booster	16–24 months Measles and Rubeola−2, DPT, OPV	Lower lateral incisor, upper and lower first molar, upper and lower canine	Fluoride varnish Oral health education to parents Early diagnosis of enamel defects for WSL.
		Second molar screening	Seal deep fissures Early diagnosis of enamel defects

#### Early diagnosis is the key

One of the primary ways to achieve the SDG3 on health indirectly relies on early diagnosis of the disease entities. Hence efforts to diagnose ECC early becomes a priority. Enamel hypoplasia or Enamel defects (EH/ED) emerging as the primary risk factor for ECC, permits a window of opportunity to diagnose them as soon as the tooth erupts into the oral cavity. Early diagnosis or identification of these changes on the enamel surface and timely interventions plays a significant role in successful management of ECC (15). With early diagnosis and minimally invasive treatment approaches, it is now possible to remineralize (with fluoride varnishes) or stabilize the carious lesions [with silver diamine fluoride (SDF)] (20–22). Therefore, awareness of oral health and hygiene practices, including early dental visits, are critical for preventing negative consequences of ECC.

Dissemination of the recent evidence on EH/ED being the primary risk factor for ECC needs to be disseminated widely to the dental, medical and primary health care workers. Simple screening and drying the tooth surface soon after its eruption will help the health care workers to identify the earliest changes of ECC. This also raises a need to create self-reporting tools useful for the parents/caretakers to identify these early changes on the enamel.

#### MAAAC charts

The MAAAC charts are a series of charts developed at the “Center for Early Childhood Caries Research (CECCRe)” to educate primary health care providers, parents, and caretakers. These charts illustrate the various early patterns of enamel defects (demarcated and diffuse opacities on various maxillary teeth) as a collage of pictures organized as incisors and canines. Thus, these charts can be practical, accessible tools for primary healthcare workers to match and report the early signs of ECC [MAAAC charts for self-diagnosis of early signs of ECC, unpublished data]. The preliminary results have been very encouraging as the parents could identify, the earliest changes on the enamel and report to the dental team soon after the tooth erupts into the oral cavity. Following either self-reports or early diagnosis by the health care team, there is a need for further guidance on handling these early changes.

#### Sustained anticipatory guidance (SAG)

Delivering cost-effective sustained interventions at an early age can also be effective in preventing ECC, the principle being termed as sustained anticipatory guidance (SAG) (26). SAG can be defined as periodic or continuous guidance/support provided by the health care workers or health care professionals, to the caretakers by education, and technology thereby facilitating early diagnosis, improved oral hygiene practices, and initiate early intervention protocols if needed. SAG was first tested in a small cohort of cleft children and reported promising results (27). SAG involved the following steps: early recruitment of the participants, motivational interviewing (MI) of primary caregivers, oral health education by audio visual aids and demonstration, providing oral hygiene aids in the form of sterile gauze pieces packed in color coded envelopes for wiping the gums of children, reinforcing the same with pictorial representation on the envelopes, providing finger toothbrush and non-fluoridated toothpaste after the eruption of the first tooth and confirming the same *via* telephone calls, SMS or follow-up visits, continuous monitoring and evaluation with application of fluoride varnish wherever necessary (effect of sustained interventions). This type of sustained interventions with periodic follow up and reinforcement could therefore be highly effective in early diagnosis and prevention of ECC (27).

#### Preventive strategies

Management of ECC involves prevention, remineralization and arrest of carious lesions (14). These can be achieved by use of various minimally invasive techniques. Less technique sensitive procedures are now possible due to the emergence of newer materials (10, 26, 28–33). These techniques are less invasive, surpassing the use of local anesthetic agents and are hence child-friendly. Thus, application of fluoride varnish, silver diamine fluoride (SDF) and glass ionomer sealants can be carried out by primary care teams (15, 30).

#### Awareness among parents and caregivers

Early intervention protocols for ECC can allow precise, easy instructions for parents and caretakers. These protocols could be followed in early infancy with few erupted teeth showing signs of non cavitated lesions (in the forms of a line or a patch on one or more tooth surfaces) and minimal or early-stage cavitation of one or more surfaces. During the first consultation, the clinical appearance of the white spot lesions and the role of plaque in the demineralization of tooth surfaces are explained to the parents. If appropriate preventive measures are not taken, the possibility of developing a full-blown ECC is also put forward to them. The consequences of severe ECC are described to them with the possibility of an intervention in the hospital under general anesthesia. Counseling regarding diet, oral hygiene measures, fluoride adequacy and the need for frequent recall to monitor the progress of the ECC is also elucidated.

Regarding diet, the importance of frequency of refined carbohydrates intake is enumerated, and the need to clean the infant's teeth after every meal or intake is stressed. The need for assistance in performing oral hygiene measures (mother or father brushing the child's teeth) is emphasized. In the presence of EH/ED, fluoride varnish applications (fluorprotector) are recommended 2–4 times in 2 months interval. If the parents take appropriate care and the professional advice is followed meticulously, ECC can be arrested at an early stage. Periodic application of fluoride varnish has been proven to aid in preventing the development of new lesions and in remineralization of white spot lesions (26, 29, 31) (Table 2).

**Table 2:** SDG targets of ECC and their implementation.

**SDG Target**	**Implementation of SDG Targets**
Prevention of ECC	First dental visit Sustained anticipatory guidance (SAG) Early interventions Prenatal oral health care Parental education sealants
Remineralization of White Spot Lesions	Fluoride varnish Early interventions protocol
Arrest of caries lesions	Silver diamine fluoride ART

#### Health education and community engagement for the prevention of early childhood caries

**Figure 1:**
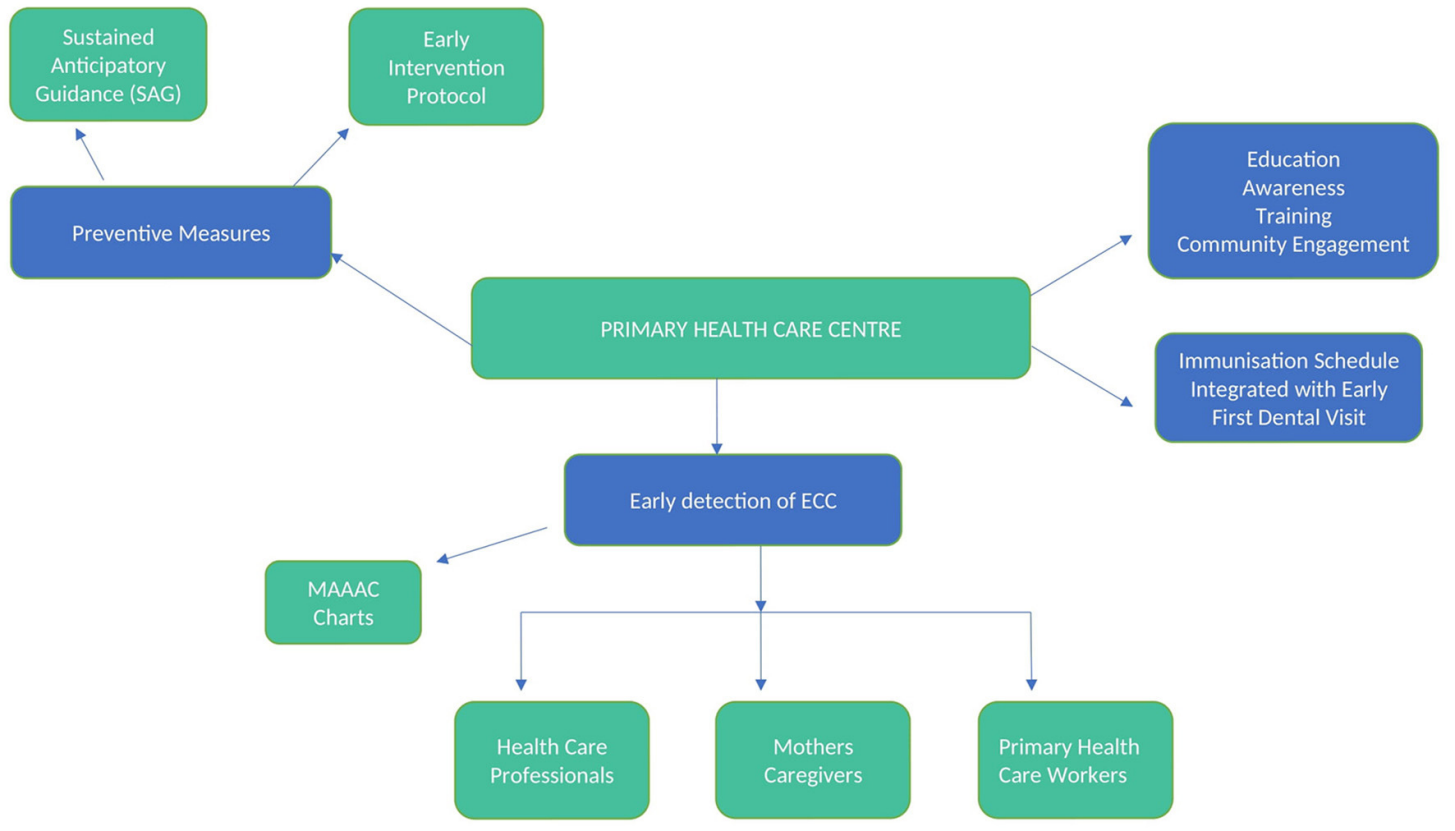
Illustrates possible integration of oral health services into primary health care centers.

It is crucial to analyze the reasons for unfavorable behaviors, such as poor oral hygiene and intake of free sugars, which are deemed primary risk factors of ECC (14). Socially disadvantaged people, such as those with low socioeconomic status and belonging to ethnic minorities, have higher rates of ECC (34). AAPD thus addresses and emphasizes the role of social determinants in poor oral health in children (35). Social Determinants of Health (SDH) are defined by the World Health Organization as “the conditions in which people are born, grow, work, live, and age, and the wider set of forces and systems shaping the conditions of daily life” (34). Thus, SDH works on improving social conditions to envision enhanced health outcomes in vulnerable populations (35). The multi-level conceptual model by Fisher-Owens demonstrates the various biological, social and environmental factors that influence the child's oral health (36). These factors highlight the need for health education and community engagement in preventing early childhood caries and thus improving children's oral health. Since a child's chief source of learning about health lies with the family (37, 38), it is prudent to create oral health awareness and increase the mindfulness about prevention of ECC among parents. Dental health education of mothers through home visits was reported to positively impact their children, resulting in better oral health for the Latter (39). Oral health education delivered to pregnant women also had a beneficial outcome on ECC prevention (40). Apart from the family-level influences, it is also necessary to address the community-level influences, such as caregivers and nursery staff at kindergarten school, health personnel who have a significant role in impact on the health of young children (14). They may be instrumental in carrying out preventive strategies for ECC, such as imparting healthy diet habits, encouraging proper tooth brushing and promoting fluoride administration (41, 42). The use of fluorides for children and oral health education for teachers and children was associated with reducing dental caries (39). Thus, preschool teachers must understand health and risk factors (43). The policies and interventions thus drafted should be inclusive of the SDH. Another importance of reaching the public is through the way of mass communication. This could be achieved by using media (television and radio), books, pamphlets, flyers, posters, emails, and SMSs to improve parental and caregiver knowledge of child oral health (44). A schematic framework has been outlined in Figure 1.

### Amalgamating oral and general health for better patient care

#### Bridging the gap between oral and general health

In 2011, the UN Political Declaration on Prevention and Control of NCDs first recognized oral diseases as a significant health burden (45, 46). This declaration was the turning point and a step forward for NCD and the oral health community in recognizing oral health and general health as one (45, 46). The WHO then advocated, Health in all Policies (HiAP) as an essential strategy that supports health in all policies. As evidence affirms common risk factors between oral diseases and NCDs, including oral health to HiAP approaches become inevitable. The FDI Vision 2020: Shaping the future of oral health at the FDI World Dental Congress (WDC) in Hong Kong, China is a marked shift in FDI's focus from a treatment-based approach to a rights-based approach to what oral health is. This publication highlights oral health as an integral component of good health. In 2020, the Member State and recommendations of the board's office and its Director-General recommended the inclusion of oral health into the 2030 Agendas (47).

The next question to consider is, “How do we bridge this gap between oral and general health?” There may be an implicit number of theories and visions from various experts on how oral and general health can be integrated, but the translation of theories to practice is critical. Furthermore, this translation requires a deeper understanding of healthcare professionals of how oral health is a part of general health. This is also essential for advocacy purposes. Therefore, the next logical question is whether it is possible to deliver a clear, precise explanation of oral health that will foster communication and support broader advocacy efforts between general health and oral health key personnel. A group of 22 experts, including the FDI-International Consortium for Health Outcomes Measurement (ICHOM) team, focused on producing a tool for measuring oral health. The team identified the key domains that fit under the physiological and psychosocial dimensions of oral health based on a series of Delphi-type consultations (Table 3). This tool can thus be an excellent example of how oral health related outcomes could be used to measure oral health related well-being, which can be linked to general well-being of an individual.

**Table 3:** The key domains for measuring the physiological and psychosocial dimensions of oral health.

**Physiological**	**Psychosocial**
Ability to eat	Overall patient satisfaction (consequential upon some physiological elements)
Chewing	Participation in life activities/social interactions
Food alteration	Emotional well-being (embarrassment/shame, anxiety/fear)
Pain-discomfort	Aesthetic satisfaction
Ability to sleep	Lost productivity
Speaking/phonetic impairment	Self-esteem, confidence

**Figure 2:**
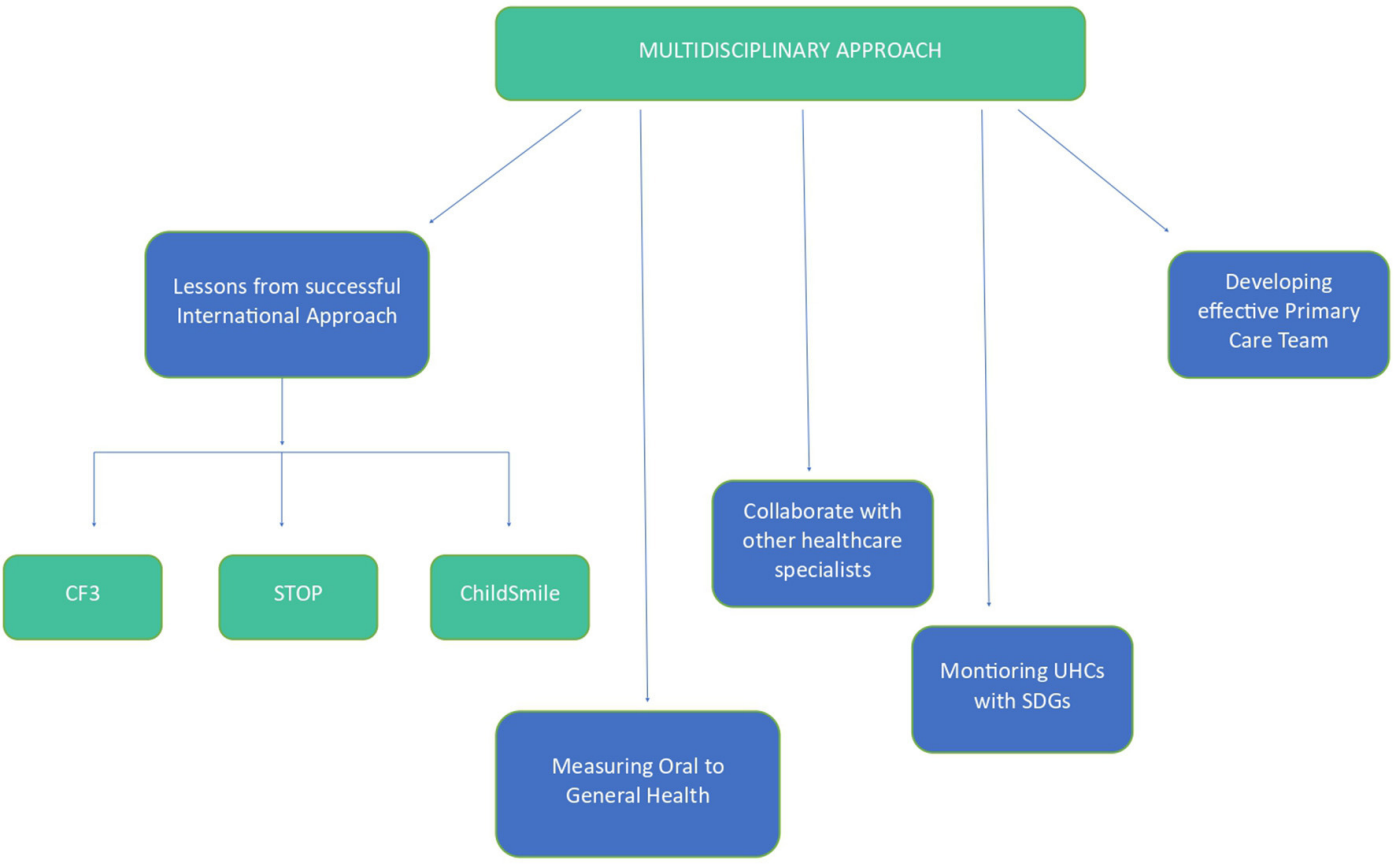
Possible Oral Health related linkage to 17 SDGs.

In 2016, FDI World Dental Congress in Poznan, Poland, proposed a definition of oral health as “multi-faceted and includes the ability to speak, smile, smell, taste, touch, chew, swallow and convey a range of emotions through facial expressions with confidence and without pain, discomfort and disease of the craniofacial complex”(48, 49). Later, this definition was adopted by an overwhelming majority at FDI's General Assembly due to the three critical elements highlighted: disease and condition status, physiological function, and psychosocial function. Therefore, the pre-eminent focus of the definition of oral health is the disease and condition status. A schematic framework of possible ways to integrate general health with oral health and the 17 sustainable development goals has been depicted in Figure 2. This framework comprises of various broad ideas and strategies. These need to be developed and customized for each country keeping in mind their demographics and cultural background.

### Multidisciplinary approach collaborating with wide range of healthcare workers

#### Developing effective primary health care teams

Children aged under five or six years are more often seen by primary care teams and less often by oral health professionals in most countries for the purpose of for vaccinations or consultation for systemic health problems. Such primary care teams who are already trained to deliver a wide range of services (e.g., child immunization, family planning, health promotion) and to treat minor conditions and injuries, have the educational background and clinical skills needed to learn about oral health promotion and control of ECC. WHO's guidelines on health policy and system support optimize community health worker programmes (14). The WHO developed robust training programmes for community health workers to acquire core competencies like i., service promotion, ii. to identify family health, risk and social health iii. to integrate work activities and the role of community health workers, including referral for health care, iv. collaborative work within primary care teams, tracking patients, surveillance, monitoring of community diseases, data collection and analysis; v. providing psychosocial support; skills related to maintaining patient confidentiality, community engagement, mobilization and personal safety (14, 50).

**Figure 3:**
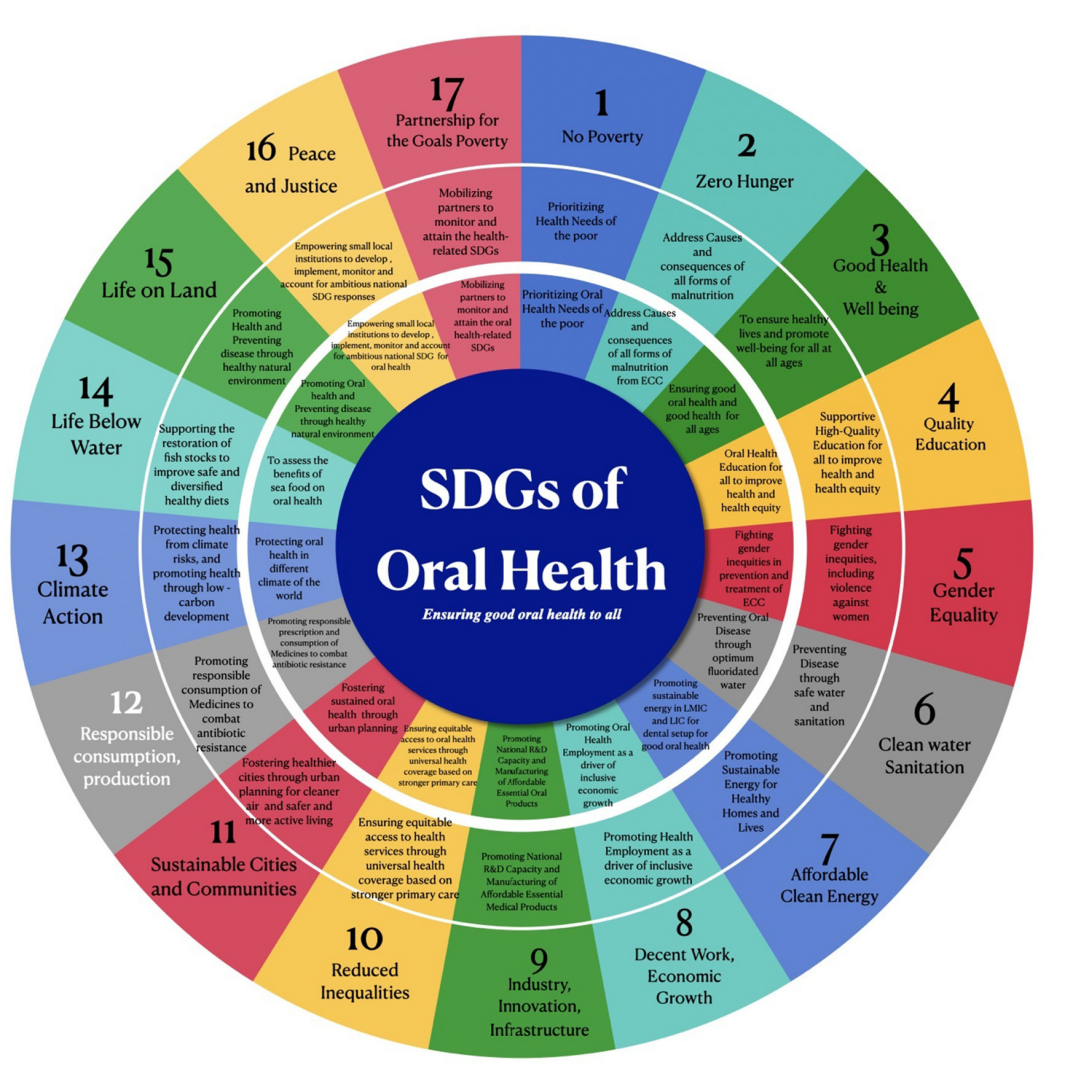
Illustrates multidisciplinary approaches and possible collaboration with wide range of healthcare workers.

**Figure 4:**
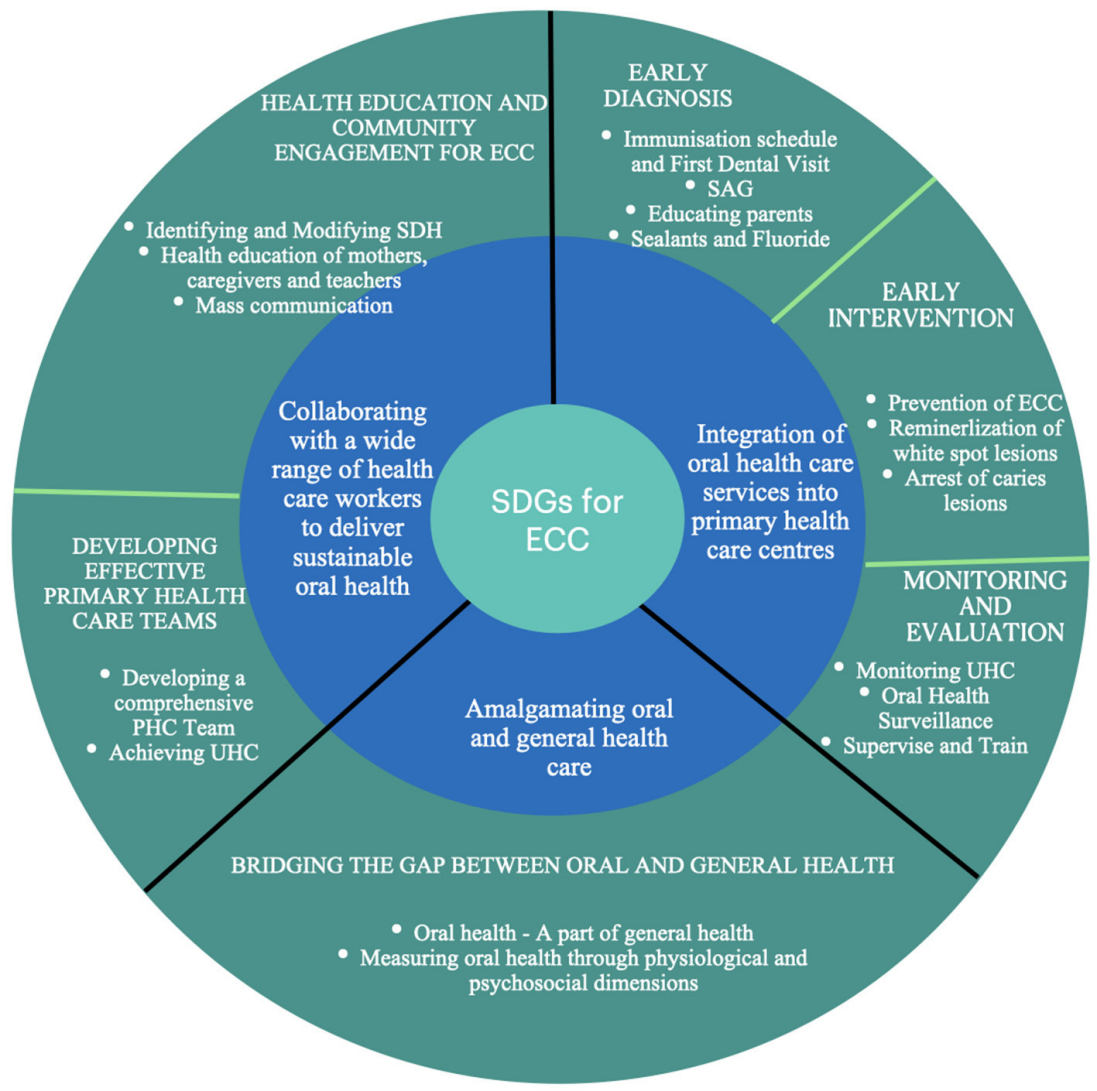
Proposed SDGs for ending ECC.

Xiao et al. in (51) reported that children whose mothers received prenatal oral health education had a reduced incidence of ECC. Educating pregnant mothers through their obstetricians can help women maintain good oral health and instill good oral hygiene practices and dietary behaviors (51, 52), which can be passed on to their children. Also, knowledge and awareness of ECC and its effect on general health should be emphasized to pediatricians who have routine access to infants and children (See Figures 3, 4).

#### Lessons from successful international approaches

##### Cavity Free at Three (CF3)

Cavity Free at Three (CF3) is a state-wide Oral Health Promotion (OHP) training program of Colorado (53) that was developed to teach medical and dental health care providers regarding oral examination, caries risk assessment, oral health instructions, dental referrals and Fluoride Varnish Application (FVA). This program proved to reduce the incidence of caries in children who received four or more FVAs by the age of 3 years (53, 54). Similar results were reported by Pahel et al. using Into the Mouth of Babe (IMB) program in children who received at least four FVAs from medical providers (55).

##### Smile train oral health program (STOP)

In addition, Smile Train Oral Health Project or STOP was developed using the SAG principles. This was piloted and tested at the Center for Early Childhood Caries Research (CECCRe). Through this program, infants with cleft lip and/palate are recruited during their early visits for lip repair to the Smile Train unit. These babies with a cleft are then provided SAG to help them grow cavity-free [Smile Train Oral Heath Project (Ongoing project)]. The phase 1 of the project achieved 69.7% caries reduction (27). The phase 2 of the project is ongoing at this point of time with 70 plus children recruited into the program.ca.

##### Collaboration with other healthcare specialties

Adopting such programs to educate obstetricians, pediatricians, physicians, physician assistants, nurses, and medical assistants can be an effective method of preventing and early intervention of ECC. Thus, the PHC team should comprise medical and dental health care providers comprising obstetricians, pediatricians and physicians, cleft care teams, nurses, midwives, kindergarten and preschool teachers, and anganwadi (in India) and social workers (Table 4).

**Table 4:** Primary health care team.

**PHC team member**	**SDG targets assigned**
Gynecologists and Obstetricians	Prenatal oral health education
Pediatricians, Physicians and Cleft care teams	Early Diagnosis, Early Intervention, Oral Health Education
Nurses, mid-wives	Oral Health Promotion services like FVAs, Early Interventions
Kindergarden and preschool teachers, anganwadi workers and social workers	Oral Health Education, Regular dental visits

#### Monitoring universal oral health coverage across nations within SDGs

Oral health coverage is the goal that all people receive essential oral health services effectively and is central to the oral health-related targets of SDGs (37). The broader scope of SDGs is to ensure the availability of a wide range of services for oral health promotion, prevention, treatment, and care, including rehabilitation and palliation (2, 37, 56). The establishment of tracer indicators to represent overall oral health coverage of essential services is recommended (56). Integrated surveillance and monitoring of clinical evidence, community programs and impact evaluation are essential to advocate conceptions, fine-tuning and practical strategies (11). Despite numerous efforts from the World Health Organization, periodic monitoring and evaluation of regional oral health policies have not been adopted in many LMICs. Supporting approaches such as “supervise and train,” which fosters flexibility in who does what, including remote supervision systems, must be integrated to achieve significant results (11, 56).

The WHO proposed, STEPwise approaches such as “WHO Oral Health Surveys Basic Methods” for oral health surveillance that could be adapted to local and national settings.

Step 1—involves assessment of oral conditions and risk factors by one's self, which in turn facilitates effective surveillance of ECC programs. The WHO Oral Health Surveys tool also facilitates the collection of information by self-reporting of dental caries, painful teeth, modifiable risk factors such as consumption of sugars and dietary patterns, oral hygiene, quality of life, and socioeconomic and environmental conditions. This tool reflects suitable core components of a questionnaire and allows self-interview. Similarly, the clarity of questionnaires has been designed for primary school teachers engaged in oral health education of young children (14).

Step 2—Clinical oral health data is collected in step 2. ECC Lesions are recorded using a WHO oral health assessment form. involves the collection of clinical oral health data. Step 2 also allows using photographs for caries assessment in countries with a shortage of oral health professionals. However, WHO insists on well-trained examiners to make reliable clinical judgements (11, 14).

Thus, it is peremptory that existing oral health promotional programs exclusive to ECC prevention are evaluated periodically. Furthermore, the involvement of preschool children in oral health surveillance programs globally and the use of mHealth applications such as “Smart Systems of Disease Management (SSDM)” can allow easy and systematic monitoring of SDGs (57).

#### Existing national approaches toward SDG and oral health

National-level health missions, infant immunization programs, and pregnant women's health programs carried out on a large scale in most countries, if integrated with Oral health/ECC and SDGs targets, can help achieve a more holistic coverage.

##### Scotland

Childsmile is a national program for the children of Scotland, developed to improve their oral health. This also targets to reduce the disparities in dental health and increase the access to dental services. As a result of this program, it has been reported that 60% children upto the age of 7 years have no noticeable decay. It has three main elements:

###### Childsmile practice

A tailored oral health advice and preventive services is provided to infants and children through Health Visitors / Public Health Nurses and Oral Health Support Workers after the child is registered.

###### Childsmile core

All children under this scheme receives 6 dental packs which includes toothbrush, toothpaste and fluoride toothpaste until 5 years.

###### Childsmile nursery and school

Children from poor communities are being offered additional fluoride varnish applications. This is provided by trained staff under NHS Highland's Salaried General Dental Service (58).

##### India

“Mission Indradhanush (MI) 4.0,” a flagship scheme, intensified in 2022 and aims to achieve 95% of immunization coverage in children. This program conducts head count survey in all districts across the country for identifying children and pregnant mothers who may have been missed out in earlier surveys. This program also allows “Flexible session timings” and “On demand vaccination timings” to ensure better turn-out of community (59). Another scheme called “Universal Immunization Programme (UIP)” is one of the most extensive public health programmes targeting close to 2.67 crore newborns and 2.9 crore pregnant women annually (NHM) (51). Integration of early dental visits with such national schemes can escalate coverage to rural pockets across nations. We propose the integration of oral health and general health aligned with the SDGs. This approach will facilitate dialogue between oral and general health practitioners focusing on tailored SDGs integration.

An important initiative called Rashtriya Bal Swasthya Karyakram (RBSK) was introduced under Nation Rural Health Mission (NHM) which aims at early identification and intervention for children from birth through 18 years of age, who will be managed at the District Early Intervention Centers (DIEC). Although, dental conditions are included under the RBSK scheme, the availability of pediatric dentists at DIECs remain sparse. Specific guidelines on early diagnosis and prevention of caries need to addressed in this scheme in future. The principle of Sustained Anticipatory Guidance developed by the Center for Early Childhood Caries Research (CECCRe) has shown promising results in prevention of caries in cleft children (SAG) (27, 60, 61).

##### Taiwan

The concept of a government run insurer with a single-payer insurance system was first established in Taiwan in 1995, to ensure health coverage to all citizens. This health insurance system provides topical fluoride application for all children under 6. In addition, pit and fissure sealant are used to seal first permeant molars along with oral prophylaxis and oral mucosal screening for children above 12 years. Thus, universal national health insurance facilitates nation-wide oral care (62).

##### Australia

The National Oral Health Plan 2014–2024 is a blueprint that ensures healthy teeth and mouths among Australian children and adults across different sectors and jurisdictions (63).

##### Brazil

The Smiling Brazil National Oral Health Program (NOHP) was launched in Brazil with the vision to prevent ECC. The model developed to achieve this involved collective and individual educational-preventive actions among pregnant women. This created an opportunity for the establishment of early dental homes for infants. This program also utilized vaccination campaigns to introduce good oral health habits for pregnant mother and newborn infants. Group activities and home visits for early identification and referral of high risk children was also another crucial element of this program (64, 65).

##### China

The National Program for Chronic Disease Control and Prevention (2017–2025) launched agendas and action plan for the Healthy China 2030. This program included the following strategies for oral health promotion (1) promoting oral health education in kindergarten, primary and middle schools. (2) developing oral health-related techniques and instruments. (3) promoting early intervention in community health service centers and township hospitals. (4) integrating oral examination into the regular physical examination. (5) developing a personalized interventions for children and the elderly, which focus on dental caries and periodontal disease management; and (6) providing topical fluoride, pit and fissure sealing and other oral health-care measures to reduce the caries prevalence rate to below 30% by 2025 (66, 67).

##### Hong Kong

In 2019, the Faculty of Dentistry at the University of Hong Kong started a “Jockey Club Children Oral Health Project” (JCCOHP), supported by The Hong Kong Jockey Club Charities Trust. This large-scale school-based oral health project aims to screen all preschool children for ECC (68). Furthermore, the program also provides early interventions for ECC, using SDF along with conducting awareness programs for parents and teachers (27).

##### Thailand

In Thailand, the Bureau of Dental Health focuses on the primary prevention of dental caries, and promotes reduction of sugar consumption through the “Sweet Enough Network.” This program, started in 2002 which was later adapted as a national policy to stop added sugar in infant formulas in 2006. This policy played a pivotal role in the reduction of ECC prevalence. Another program was launched by the Ministry of Public Health called “Tooth-brushing on the first tooth eruption with appropriate fluoride toothpaste.” This campaign aims to enhance community participation along with providing hands-on training for the mothers or caregivers on how to brush the baby's first tooth (69, 70).

##### United Kingdom

The National Health Service (NHS) provides free oral care in England and other devolved nations such as Scotland, Wales, and Northern Ireland (71).

##### The way forward

Although the work on integrating oral health into general health is underway, the introduction of robust SDGs and their targets for ending ECC will pave the way for using oral health outcome measurements as the basis for remuneration schemes. The monitoring and evaluation (M&E) frameworks for measuring the progress of national health policies, strategies, SDGs and core health indicators have been established across nations. How well “oral health” M&E fits into this framework solely depends on four critical steps:

1. Inclusion of oral health under SDG3 or health goal,

2. Establishment of childhood caries under non-communicable disease (NCD),

3. Amalgamation of oral health with UHC,

4. Embedding oral health into all health policies.

Furthermore, linking potential SDGs to the health linkage with 17 SDGs could accelerate the process across the globe (Figure 4). National-level health missions, infant immunization programs, and pregnant women's health programs carried out on a large scale in most countries, if integrated with ECC SDGs targets, can also help achieve more excellent coverage.

### Technology-driven teledentistry/mhealth in ending ECC

The term digital health is rooted in e-health and allows the employment of consistent and effective forms of healthcare delivery for underserved populations. In 2018, the World Health Assembly Resolution on Digital Health recognized the value of digital technologies in contributing to the advancement of Universal Health Coverage (72). The American Academy of Pediatric Dentistry (AAPD) endorses teledentistry (a part of teleHealth) as an excellent way of improving access to care for children (73). Broader sets of recommendations for digital health include transmission of patient records, a real-time live video involving patient-doctor interaction, remote monitoring of patients, and mobile phone technologies, which is termed mHealth (74). Mobile Health (mHealth), a subset of e-health, allows mobile technologies for health. The extensive use of mobile phone technologies in health has led to significant translation across the globe. In Low- and Middle-income countries (LMICs), the growth of mobile phone networks with more incredible data transmission speeds has allowed for the transformation of the accessibility, delivery and management of health care services (74). Amid the heightened interest, digital health must be developed in tune with the SDG targets and only when oral healthcare demands can be met. The set core recommendations of digital health can include video consultation, SAG, ECC timeline, AI-assisted picture-based applications for early diagnosis of ECC, diet monitoring, and a repository of dental records. Furthermore, integrations of oral digital health with general digital health can leveraged to enhance the success of UHC. Appointment reminders using Short Messaging Services (SMS) can be effectively used in the periodic follow-up of pediatric patients, which can aid in the early diagnosis of white spot lesions. Information on preventive strategies such as fluoride application, Sealants and SDF can be sent *via* mobile services to parents of young children in rural regions. Oral Health promotion and awareness of ECC among parents, caregivers and preschool teachers could be provided through mHealth. Reminders on reducing sugar intake can act as a reinforcement measure. Automated daily SMS in regional languages has proven to be an effective strategy to sustain health education among parents. Such approaches have revealed significant results in ECC prevention among children with a cleft.

### Challenges underway

#### Lack of financing

The importance of consolidating oral health within primary health care systems and universal health coverage programmes has been described in the Lancet Issue on Oral Health (2019) (75). Coverage on dental benefits by health system has been restricted in most countries (76, 77). Limited coverage on dental care could cause inequalities in access to dental health services, leading to disparities in oral health (75). Research on variations in coverage and access to dental care remains limited (77–79).

#### Instability of resources

The year 2022 has faced the sharpest economic slowdown in past eight decades with rising inflation, food insecurity, war and the continued negative impact of Covid−2019 (22). The war in Ukraine has caused global financial conditions to be tightened, mostly affecting Eastern Europe and Middle East countries with close ties to Russia. Also the International Monetary Fund (IMF) chief has reported Srilanka's economic crisis to be a warning to several other countries with high debt. Also the sharp rise in prices of commodities is anticipated to increase the inflation pressure (80). Such instances could cause instability of resources in low and lower-middle income countries. This poses a challenge in mobilizing financial resources toward dental health services.

#### Lack of human resources

An increase in the number of dental schools has led to an increase in the number of dentists. But sadly, the availability of dentists in rural areas is still sparse. Also, in LICs, very few dental schools exist and hence the availability of dental personnel is extremely low (11).

#### Lack of proper insurance

Dental Insurance schemes could play a major role in treating patients with low socio-economic status, who would otherwise ignore dental services. In the United States of America, Medicaid provides health insurance to most population including the low-income groups. It also provides dental insurance for children to relieve pain and infection, restoration of teeth and maintenance of dental health [Medicaid.Gov]. In United Kingdom (UK), insurance is provided by the National Health Service (NHS). However, these are High—Income countries. In India, Arogya Finance provides medical and dental loans. However, presence of dental insurance in low and middle income countries still remain a far-fetched reality.

Despite vital initiatives and the development of strategic frameworks for ending ECC (WHO, FDI), the success of these frameworks is possible only if they are in practice. Once launched, monitoring and sustaining these goals are the biggest challenge. Factors such as politics, governance, national health schemes, education, regional oral health leaders, trained personnel, socio-demographic index etc., can constrain the implementation of these solution-based frameworks. Nonetheless, oral health leaders must demonstrate and prove the impact of oral health on general health in a child. Overcoming these fundamental challenges can be elusive if not proven.

## Conclusion

With the current knowledge of ECC and its effects on the child's general health, it is imperative to set up long-term sustainable goals, which could be beneficial for the pediatric population. Shifting the focus from treatment minimally invasive, preventive and to patient-driven is critical to achieve an exponential outcome from such strategies. The culmination of oral health with NCD, UHC and SDG3 are the key steps. Raising awareness at the global, national and regional levels through benchmark research will mitigate the ECC burden in societies. Early Interventional Protocol and principles of SAG need to be widely disseminated. Political mandate, proper governance, using the common risk factor approaches and the alignment of ECC SDG targets with emerging political opportunities can further aid to remove regional disparity. Surveillance and monitoring of demographics and health-related indicators in the SDGs era will aid in attaining sustainability. Tailored cutting-edge mHealth services need to be curated to achieve these sustainable targets across nations.

## Author contributions

AS, AJ, and MS contributed to design, acquisition, analysis, interpretation, drafted manuscript, critically revised the manuscript, gave final approval, and agrees to be accountable for all aspects of work ensuring integrity and accuracy. RA contributed to design, drafted manuscript, contributed to interpretation, critically revised the manuscript, gave final approval, and agrees to be accountable for all aspect of work ensuring integrity and accuracy. AS and AJ drafted manuscript, critically revised manuscript, gave final approval, and agrees to be accountable for all aspects of work ensuring integrity and accuracy. SP, TW, and MS critically revised manuscript for important intellectual content, gave final approval, and agrees to be accountable for all aspects of work ensuring integrity and accuracy. MS contributed to conception and design, contributed to acquisition, contributed to analysis, contributed to interpretation, drafted the manuscript, critically revised the manuscript, gave final approval, and agrees to be accountable for all aspect of work ensuring integrity and accuracy. PM and MD contributed to acquisition, critically critically revised the manuscript, gave final approval, and agrees to be accountable for all aspects of work ensuring integrity and accuracy. All authors contributed to the article and approved the submitted version.

## Conflict of interest

The authors declare that the research was conducted in the absence of any commercial or financial relationships that could be construed as a potential conflict of interest.

## Publisher's note

All claims expressed in this article are solely those of the authors and do not necessarily represent those of their affiliated organizations, or those of the publisher, the editors and the reviewers. Any product that may be evaluated in this article, or claim that may be made by its manufacturer, is not guaranteed or endorsed by the publisher.

## References

[1] UN. Transforming Our World: The 2030 Agenda for Sustainable Development. (2003). New York: United Nations. Available online at: https://sustainabledevelopment.un.org/post2015/transformingourworld (accessed April 14, 2015).

[2] IAEG-SDGs. Report of the Inter-Agency and Expert Group on the Sustainable Development Goal Indicators. (2016). New York, NY: Economic and Social Council, 2016. Available online at: http://unstats.un.org/unsd/statcom/47th-session/documents/2016-2-SDGs-Rev1-E.pdf (accessed July 15, 2016).

[3] Global regional and national incidence prevalence and years lived with disability for 328 diseases and injuries for 195 countries 1990–2016: 1990–2016: a systematic analysis for the Global Burden of Disease Study 2016. Lancet. (2017) 390:1211–59. 10.1016/S0140-6736(17)32154-2 28919117 PMC5605509

[4] Benzian H Hobdell M Holmgren C Yee R Monse B Barnard JT. Political priority of global oral health: an analysis of reasons for international neglect. Int Dent J. (2011) 61:124–30. 10.1111/j.1875-595X.2011.00028.x 21692782 PMC9374826

[5] Mathur MR Williams DM Reddy KS Watt RG. Universal health coverage a unique policy opportunity for oral health. J Dent Res. (2015) 94:3S−5S. 10.1177/0022034514565648 25710897 PMC4541093

[6] World Health Organization. Global Status Report on Noncommunicable Diseases. (2014). http://www.who.int/nmh/publications/ncd-status-report-2014/en/ (accessed 5 Sept 2017). 10.1161/STROKEAHA.115.008097 25873596

[7] World Health Organization. Non communicable Diseases Country Profiles. (2018). Available online at: https://apps.who.int/iris/handle/10665/274512 (accessed 25 March 2022).

[8] Schrijvers G. Disease management: a proposal for a new definition. Int J Integr Care. (2009) 9:e06. 10.5334/ijic.301 19340329 PMC2663707

[9] Selwitz RH Ismail AI Pitts NB. Dental caries. Lancet. (2007) 369:51–9. 10.1016/S0140-6736(07)60031-2 17208642

[10] US US Department of Health and Human Services National National Institute of Dental and Craniofacial Research US Public Health Service. Oral Health in America: Report of the US Surgeon General. NIH publication no. 00-213. Washington, DC: DHHS, NIDCR, USPHS. (2000).

[11] Watt RG Daly B Allison P Macpherson LMD Venturelli R Listl S. Ending the neglect of global oral health: time for radical action. Lancet. (2019) 394:261–72. 10.1016/S0140-6736(19)31133-X 31327370

[12] FDI. World Dental Federation - Vision 2030. (2021). Available online at: https://www.fdiworlddental.org/vision-2030-delivering-optimal-oral-health-all (accessed 13 March 2022).

[13] Folayan MO El Tantawi M Aly NM Al-Batayneh OB Schroth RJ Castillo JL. Association between early childhood caries and poverty in low and middle income countries. BMC Oral Health. (2020) 20:8. 10.1186/s12903-019-0997-9 31906944 PMC6945445

[14] National Advisory Committee on Rural Health Human Services (2004). The 2004 Report to the Secretary: Rural Health and Human Service Issues. Washington, DC: USDHHS. (2004). Available online at: https://www.hrsa.gov/sites/default/files/hrsa/advisory-committees/rural/reports-recommendations/2004-report-to-secretary.pdf. (accessed July 15, 2021).

[15] World Health Organization. (2019). Ending Childhood Dental Caries: WHO Implementation Manual. Available online at: https://apps.who.int/iris/handle/10665/330643 (accessed 25 April 2022).

[16] National Institute of Dental Craniofacial Research (U.S.), and United States (2000). Oral Health in America: A Report of the Surgeon General. Rockville, Md.: U.S. Public Health Service, Dept. of Health and Human Services. Available online at: https://www.cdc.gov/oralhealth/publications/federal-agency-reports/sgr2000_05.htm (accessed 23 April 2022).

[17] Peres MA Macpherson LM Weyant RJ. Oral diseases: a global public health challenge. Lancet. (2019) 394:249–60. 10.1016/S0140-6736(19)31146-8 31327369

[18] Heilmann A Tsakos G Watt RG. Oral health over the life course. In: Burton-Jeangros C, Cullati S, Sacker A, Blane D, editors. A Life Course Perspective on Health Trajectories and Transitions. Cham (CH): Springer (2015). 27683931

[19] FDI World Dental Federation (2015). The Challenge of Oral Disease—A Call for Global Action. The Oral Health Atlas. 2nd ed. Geneva: FDI World Dental. Available online at: https://www.fdiworlddental.org/resources/publications/oral-health-atlas/oral-health-atlas-2015 (accessed 25 September 2020).

[20] World Health Organization. Primary Health Care. World Health Organization (2018). Available online at: https://www.who.int/news-room/fact-sheets/detail/primary-health-care (accessed 14 April 2022).

[21] World Health Organization the World Bank USAID (2015). Health Measurement and Accountability Post 2015: Five-Point Call to Action. Available online at: http://www.who.int/hrh/news/2015/5-point-call-to-action.pdf?ua=1 (accessed 24 March 2022).

[22] World Health Organization the World Bank USAID (2017). The Roadmap for Health Measurement and Accountability. Available online at: http://www.who.int/hrh/documents/roadmap4health-measurement_accountability.pdf?ua=1 (accessed 20 January 2017).

[23] Baker SD Lee JY Wright R. The Importance of the Age One Dental Visit. Chicago, Il: Pediatric Oral Health Research and Policy Center. American Academy of Pediatric Dentistry (2019).

[24] Banerjee A Doméjean S. The contemporary approach to tooth preservation: minimum intervention (MI) caries management in general practice. Prim Dent J. (2013) 2:30–7. 10.1308/205016813807440119 24340496

[25] Fejerskov O. Concepts of dental caries and their consequences for understanding the disease. Community Dent Oral Epidemiol. (1997) 25:5–12. 10.1111/j.1600-0528.1997.tb00894.x 9088687

[26] Marinho VC Worthington HV. Walsh T, Clarkson JE. Fluoride varnishes for preventing dental caries in children and adolescents. Cochrane Database Syst Rev. (2013) (7):CD002279. 10.1002/14651858.CD002279.pub2 23846772 PMC10758998

[27] Abirami S Panchanadikar N Muthu MS Balasubramanian S Murthy J Mohan A. Effect of sustained interventions from infancy to toddlerhood in children with cleft lipand palate for preventing early childhood caries. Caries Res. (2021) 22:1–9. 10.1159/000517210 34293739

[28] Weyant RJ Tracy SL Anselmo TT Beltrán-Aguilar ED Donly KJ Frese WA. Topical fluoride for caries prevention: executive summary of the updated clinical recommendations and supporting systematic review. J Am Dent Assoc. (2013) 144:1279–91. 10.14219/jada.archive.2013.0057 24177407 PMC4581720

[29] Lenzi TL Montagner AF Soares FZ de Oliveira Rocha R. Are topical fluorides effective for treating incipient carious lesions? A systematic review and meta-analysis. J Am Dent Assoc. (2016) 147:84–91. 10.1016/j.adaj.2015.06.018 26562737

[30] Fejerskov O. Changing paradigms in concepts on dental caries: consequences for oral health care. Caries Res. (2004) 38:182–91. 10.1159/000077753 15153687

[31] Gao SS Zhang S Mei ML Lo EC Chu CH. Caries remineralisation and arresting effect in children by professionally applied fluoride treatment: a systematic review. BMC Oral Health. (2016) 16:12. 10.1186/s12903-016-0171-6 26831727 PMC4736084

[32] Marinho VC Higgins J Logan S Sheiham A. Fluoride toothpastes for preventing dental caries in children and adolescents. Cochrane Database Syst Rev. (2003) 1:CD002278. 10.1002/14651858.CD002284 12535435 PMC8439270

[33] Marinho VCC Higgins JPT Sheiham A Logan S. Combinations of topical fluoride (toothpastes, mouthrinses, gels, varnishes) versus single topical fluoride for preventing dental caries in children and adolescents. Cochrane Database Syst Rev. (2004) 1:CD002781. 10.1002/14651858.CD002781.pub2 14973992 PMC6999808

[34] Kim Seow W. Environmental, maternal, and child factors which contribute to early childhood caries: a unifying conceptual model. Int J Paediatr Dent. (2012) 22:157–68. 10.1111/j.1365-263X.2011.01186.x 21972925

[35] American Academy of Pediatric Dentistry. Policy on social determinants of children's oral health and health disparities. In: The Reference Manual of Pediatric Dentistry. Chicago, Ill: American Academy of Pediatric Dentistry. (2021). p. 28–31.

[36] Fisher-Owens SA Gansky SA Platt LJ Weintraub JA Soobader MJ Bramlett MD. Influences on children's oral health: a conceptual model. Pediatrics. (2007) 120:e510–20. 10.1542/peds.2006-3084 17766495

[37] Brickhouse TH. Family oral health education. Gen Dent. (2010) 58:212–19. 20478801

[38] Hooley M Skouteris H Boganin C Satur J Kilpatrick N. Parental influence and the development of dental caries in children aged 0–6 years: a systematic review of the literature. J Dent. (2012) 40:873–85. 10.1016/j.jdent.2012.07.013 22842202

[39] Ammari JB Baqain ZH Ashley PF. Effects of programs for prevention of early childhood caries: a systematic review. Med Princ Pract. (2007) 16:437–42. 10.1159/000107748 17917443

[40] Henry JA Muthu MS Swaminathan K Kirubakaran R. Do oral health educational programmes for expectant mothers prevent early childhood caries?—systematic review. Oral Health Prev Dent. (2017) 15:215–21. 10.3290/j.ohpd.a38522 28674702

[41] Vann WF Lee JY Baker D Divaris K. Oral health literacy among female caregivers: impact on oral health outcomes in early childhood. J Dent Res. (2010) 89:1395–400. 10.1177/0022034510379601 20924067 PMC3123718

[42] Naidu R Nunn J Irwin JD. The effect of motivational interviewing on oral healthcare knowledge, attitudes and behaviour of parents and caregivers of preschool children: an exploratory cluster randomised controlled study. BMC Oral Health. (2015) 15:101. 10.1186/s12903-015-0068-9 26328785 PMC4556322

[43] Jürgensen N Petersen PE. Promoting oral health of children through schools–results from a WHO global survey 2012. Community Dent Health. (2013) 30:204–18. 24575523

[44] Albino J Tiwari T. Preventing childhood caries: a review of recent behavioral research. J Dent Res. (2016) 95:35–42. 10.1177/0022034515609034 26438210 PMC4700662

[45] World Dental Federation (2016). American Dental Association Health Policy Institute. Oral health and well-being in the United States. Available online at: http://www.ada.org/en/science-research/health-policy-institute/oral-health-and-well-being (accessed 24 April 2022).

[46] World Health Organization (2021). Primary Health Care. Available online at: https://www.who.int/news-room/fact-sheets/detail/primary-health-care (accessed 20 March 2022).

[47] UN. Local 2030 a global multi-stakeholder initiative to support the local-level implementation of the SDGs. (2015) (accessed 20 April 2022).

[48] Glick M Monteiro O Seeberger GK. FDI Vision 2020: shaping the future of oral health. Int Dent J. (2012) 62:278–91. 10.1111/idj.12009 23252585 PMC9374976

[49] World Dental Federation (2017). Promoting Health in All Policies and Intersectoral Action Capacities. Available online at: http://www.who.int/activities/promoting-health-in-all-policies-and-intersectoral-action-capacities (accessed 05 2022).

[50] Who Guideline on Health Policy and System Support to Optimize Community Health Worker Programmes. Geneva: World Health Organization. (2018). 30431747

[51] Xiao J Alkhers N Kopycka-Kedzierawski DT Billings RJ Wu TT Castillo DA. Prenatal oral health care and early childhood caries prevention: a systematic review and meta-analysis. Caries Res. (2019) 53:411–21. 10.1159/000495187 30630167 PMC6554051

[52] Vilella KD Fraiz FC Benelli EM Assuncao LR. Oral health literacy and retention of health information among pregnant women: a randomised controlled trial. Oral Health Prev Dent. (2017) 15:41–8. 10.3290/j.ohpd.a37712 28232973

[53] Rural Health Information Hub. Cavity Free at Three. Rural Health Information Hub. (2019). Available online at: https://www.ruralhealthinfo.org/project-examples/647 (accessed 19 March 2022).

[54] Braun PA Widmer-Racich K Sevick C Starzyk EJ Mauritson K Hambidge SJ. Effectiveness on early childhood caries of an oral health promotion program for medical providers. Am J Public Health. (2017) 107:S97–103 10.2105/AJPH.2017.303817 28661802 PMC5497886

[55] Pahel BT Rozier RG Stearns SC Quiñonez RB. Effectiveness of preventive dental treatments by physi- cians for young Medicaid enrollees. Pediatrics. (2011) 127:e682–9. 10.1542/peds.2010-1457 21357343 PMC3065140

[56] Oral health left out of global health goals. Br Dent J. (2018) 225:913. 10.1038/sj.bdj.2018.1052

[57] GBD 2015 Risk Factors Collaborators. Global, regional, and national comparative risk assessment of 79 behavioural, environmental and occupational, and metabolic risks or clusters of risks, 1990–2015: a systematic analysis for the Global Burden of Disease Study 2015. Lancet. (2016) 388:1659–24. 10.1016/S0140-6736(16)31679-8 27733284 PMC5388856

[58] Childsmile. Childsmile – Improving the Oral Health of Children in Scotland. (2014). Available online at: http://www.child-smile.org.uk/ (accessed September, 2022).

[59] Ministry of Health Family Welfare. Intensified Mission Indradhanush 4.0. (2022). Available online at: https://imi4.nhp.gov.in/assets/document/operational/IMI4.0_oprational_guidelines.pdf (accessed on 5 April 2022).

[60] National Health Mission (2014). Universal Immunization Programme (UIP). American Academy of Pediatric Dentistry. Policy on early childhood caries (ECC): Consequences and preventive strategies. The Reference Manual of Pediatric Dentistry. Chicago, Ill.: American Academy of Pediatric Dentistry. (2021). Available online at: https://nhm.gov.in/index1.php?lang=1&level=2&sublinkid=824&lid=220 (accessed in 26 April 2022).

[61] Chen J Duangthip D Gao SS Huang F Anthonappa R Oliveira BH. Oral health policies to tackle the burden of early childhood caries: a review of 14 countries/regions. Front Oral Health. (2021) 2:670154. 35048013 10.3389/froh.2021.670154 PMC8757786

[62] Huang YK Chang YC. Oral health: the first step to sustainable development goal 3. J Formos Med Assoc. (2022) 121:1348–50. 10.1016/j.jfma.2021.10.018 34732302

[63] Australian Institute of Health and Welfare (AIHW). Oral Health and Dental Care in Australia. Canberra: AIHW. (2019).

[64] Frazão P Narvai PC. Water fluoridation in Brazilian cities at the first decade of the 21st century. Rev Saude Publica. (2017) 51:47. 10.1590/s1518-8787.2017051006372 28513762 PMC5425242

[65] Castro MC Massuda A Almeida G Menezes-Filho NA Andrade MV de Souza Noronha KVM. Brazil's unified health system: the first 30 years and prospects for the future. Lancet. (2019) 394:345–56. 10.1016/S0140-6736(19)31243-7 31303318

[66] Tan X Liu X Shao H. Healthy China 2030: a vision for health care. Value Health Reg Issues. (2017) 12:112–4. 10.1016/j.vhri.2017.04.001 28648308

[67] Zhou X Xu X Li J Hu D Hu T Yin W. Oral health in China: from vision to action. Int J Oral Sci. (2018) 10:1. 10.1038/s41368-017-0006-6 29343681 PMC5944598

[68] Faculty of Dentistry The University of Hong Kong (2020). Jockey Club Children Oral Health Project. Available online at: https://www.jccohp.hku.hk/?lang=en (accessed September, 2022).

[69] Thai Health Promotion Foundation. 60 Outstanding Performances, 2001–2009. (2017). Available online at: https://dol.thaihealth.or.th/Media/Pdfview/7070f49b-978c-e711-80e3-00155d65ec2e (accessed September, 2022).

[70] Thai Bureau of Dental Health. 2018 Guideline for Proceeding of Dental Health. (2017). Available online at: http://dental2.anamai.moph.go.th/ewtadmin/ewt/dental/ewt_dl_link.php?nid=1691 (accessed September, 2022).

[71] Health Social Care Information Centre. Children's Dental Health Survey 2013. (2015). Available online at: https://files.digital.nhs.uk/publicationimport/pub17xxx/pub17137/cdhs2013-report2-dental-disease.pdf (accessed September, 2022).

[72] World Health Organization (2016). Social Determinants of Health. Available online at: http://www.who.int/social_determinants/sdh_definition/en/ (accessed September 2022).

[73] American Academy of Pediatric Dentistry. Policy on teledentistry. The Reference Manual of Pediatric Dentistry. Chicago, Ill.: American Academy of Pediatric Dentistry. (2021). p. 51–2.

[74] World Health Organization. mHealth New horizons for health through mobile technologies. Based on the findings of the second global survey on eHealth. (2011). Available online at: http://apps.who.int/iris/bitstream/handle/10665/44607/9789241564250_eng.pdf?sequence=1 (accessed 23 March 2022).

[75] Winkelmann J Gómez Rossi J Schwendicke F Dimova A Atanasova E Habicht T. Exploring variation of coverage and access to dental care for adults in 11 European countries: a vignette approach. BMC Oral Health. (2022) 22:65. 10.1186/s12903-022-02095-4 35260137 PMC8905841

[76] Schoenberg NE Ravdal H. Using vignettes in awareness and attitudinal research. Int J Soc Res Methodol. (2000) 3:63–74. 10.1080/136455700294932

[77] Allin S Farmer J Quiñonez C Peckham A Marchildon G Panteli D. Do health systems cover the mouth? Comparing dental care coverage for older adults in eight jurisdictions. Health Policy. (2020) 124:998–1007. 10.1016/j.healthpol.2020.06.015 32712013

[78] Palència L Espelt A Cornejo-Ovalle M Borrell C. Socioeconomic inequalities in the use of dental care services in Europe: what is the role of public coverage? Community Dent Oral Epidemiol. (2014) 42:97–105. 10.1111/cdoe.12056 23786417 PMC3864569

[79] Manski R Moeller J Chen H Widström E Lee J Listl S. Disparity in dental coverage among older adult populations: a comparative analysis across selected European countries and the USA. Int Dent J. (2015) 65:77–88. 10.1111/idj.12139 25363376 PMC4376582

[80] IMF. IMF Support for Low-Income Countries, February 16, Washington: International Monetary Fund. (2021). Available online at: https://www.imf.org/en/About/Factsheets/IMF-Support-for-Low-Income-Countries (accessed on 12 March, 2022).

